# Right bronchial artery aneurysm successfully managed by embolization: Report of a rare case

**DOI:** 10.1016/j.radcr.2025.03.069

**Published:** 2025-04-18

**Authors:** Tamara Aburiash, Moaath Sawalha, Yazan Giacaman, Mohammed Khader

**Affiliations:** a Department of Medicine, An-Najah National University, Nablus, Palestine; b Department of Radiology, An-Najah National University Hospital, Nablus, Palestine

**Keywords:** Bronchial artery aneurysm (BAA), Hemoptysis, Aneurysm rupture, Bronchial artery embolization (BAE), Interventional Radiology

## Abstract

Bronchial artery aneurysms (BAAs) are rare but can cause life-threatening pulmonary hemorrhage. Early diagnosis and treatment are crucial for preventing fatal complications. BAAs may be associated with inflammatory lung diseases or may occur idiopathically. Super-selective bronchial artery embolization has become the preferred therapeutic approach due to its safety and efficacy in controlling bleeding.

We present a case of a 31-year-old otherwise healthy female who experienced recurrent hemoptysis and was found to have a large, tortuous right bronchial artery aneurysm measuring 2.3 × 3.6 cm, identified via computed tomography (CT) and angiography. The aneurysm originated from the descending aorta. Given the high risk of rupture, the patient underwent right bronchial artery embolization (BAE) using Contour particles and coils, achieving a favorable outcome. Follow-up imaging is planned to ensure long-term success and prevent recurrence.

## Introduction

Bronchial artery aneurysms (BAAs) are rare, accounting for less than 1% of all aneurysms, yet they pose a significant risk of life-threatening hemorrhage [1]. BAAs can be classified as either primary (idiopathic) or secondary, often resulting from trauma, bronchopulmonary infections, pulmonary malignancies, coagulation disorders, or connective tissue diseases [2]. Chronic inflammation can lead to vascular wall weakening, increased vascularization, and tortuosity, all contributing to aneurysm formation [3].

The clinical presentation of BAAs ranges from asymptomatic cases to recurrent hemoptysis, the hallmark symptom. Additional symptoms may include chest pain or mediastinal blood accumulation. Spontaneous rupture can lead to massive hemoptysis and respiratory failure [4]. A CT angiogram is the preferred diagnostic modality, as chest X-rays have poor sensitivity in detecting aneurysms [5].

Historically, surgical treatments such as aneurysm resection or lobectomy were performed; however, interventional radiology has revolutionized management. BAE has demonstrated superior efficacy compared to surgery, offering a higher success rate with fewer complications [3,6].

This report presents the case of a previously healthy 31-year old female with an unusual presentation of recurrent hemoptysis due to a tortuous bronchial artery aneurysm, successfully managed with BAE. This case underscores the importance of timely diagnosis and treatment, as well as the efficacy of BAE in preventing life-threatening complications.

### Case presentation

A 31-year-old previously healthy female developed fever, generalized fatigue, and myalgia one week prior to admission. Initially treated for an upper respiratory tract infection, she was discharged but returned 3 days later with recurrent hemoptysis, right-sided chest pain, fever (40°C), and headache. A CT scan revealed significant findings, prompting further evaluation.

A contrast-enhanced chest CT showed a dilated, tortuous right bronchial artery arising from the descending aorta, with several adjacent dilated vascular structures at the right hilum, posterior to the lower trachea, and in the subcarinal region. The largest aneurysm, located inferior to the azygos vein, measured 2.3 × 3.6 cm. There were no filling defects in the pulmonary trunk, main pulmonary arteries, or segmental branches, ruling out thromboembolism.

**Fig. 1:**
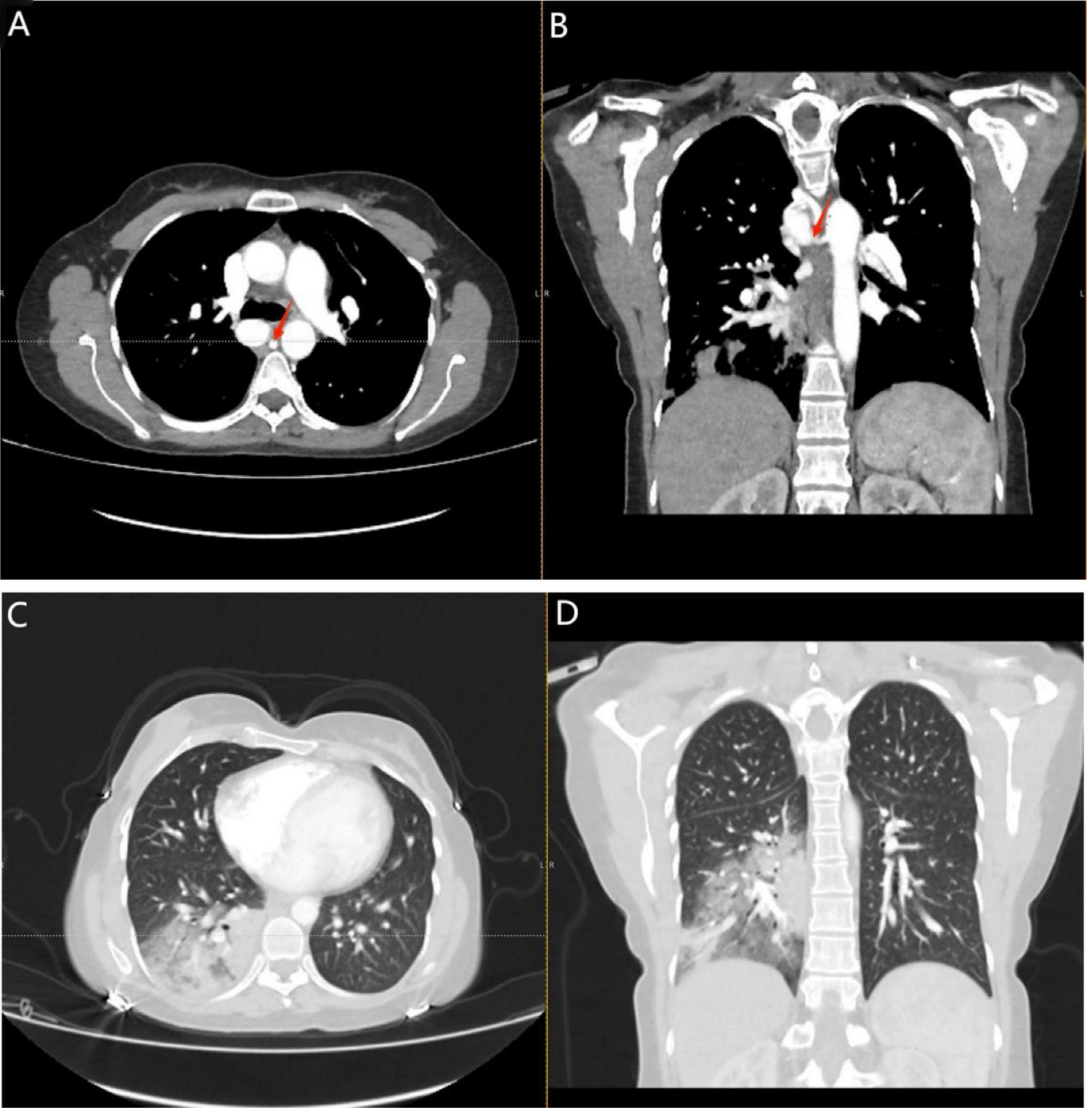
(A) Axial contrast-enhanced CT angiography of the chest showing a right bronchial artery aneurysmal dilatation measuring approximately 2.3 × 3.6 cm (red arrow). (B) Coronal contrast-enhanced CT angiography of the chest showing a right bronchial artery aneurysmal dilatation measuring approximately 2.3 × 3.6 cm (red arrow). (C) Axial noncontrast lung window CT of the chest showing Right lower zone consolidation with air bronchogram in keeping with alveolar airspace disease suggestive of pulmonary hemorrhage or infection. (D) Coronal noncontrast lung window CT of the chest showing Right lower zone consolidation with air bronchogram in keeping with alveolar airspace disease suggestive of pulmonary hemorrhage or infection.

Additional small vascular structures arising from the anterior descending aorta were noted at the left hilar region, adjacent to the left mainstem bronchus. The findings suggested a bronchial artery aneurysm or arteriovenous malformation. Right lower lobe consolidation with an air bronchogram suggested pulmonary hemorrhage or infection as shown in (Fig. 1A-D).

She presented to our hospital for further investigations. On physical examination. The patient appeared well, conscious, oriented, and alert. She was hemodynamically stable, with decreased air entry on the right side, no added lung sounds, regular heart sounds, a soft, lax abdomen, and no tenderness or distention. There were no signs of peripheral edema, lymphadenopathy, or skin lesions.

Laboratory results including hemoglobin were normal as shown in Table 1. Additionally, echocardiography was normal, ruling out infective endocarditis or cardiac-related embolic sources as potential causes. The infectious etiology was ruled out based on normal laboratory results, lack of systemic infection markers, and imaging findings that were more indicative of a vascular abnormality rather than infection-related pathology. No evidence of abscesses, necrotizing pneumonia, or mycotic aneurysms was found on imaging. Based on the clinical presentation, imaging, and laboratory findings, the patient was diagnosed with a right bronchial artery aneurysm causing recurrent hemoptysis. After a multidisciplinary, the decision was made to proceed with BAE to prevent further episodes of life-threatening hemoptysis.

**Table 1:** Laboratory investigations.

Parameter	Value	Reference range
White cell count (WBC)	10.15	4.5-11.0 ×10^3/µL
Red blood count (RBC)	4.39	4.1-5.1 ×10^6/µL
Hemoglobin (HGB)	12.8	12.0-15.5 g/dL
Hematocrit (HCT)	38.4	36.0%-47.0%
Platelet count (PLT)	238	150-400 ×10^3/µL
Neutrophil %	58.8	40%-75%
Lymphocyte %	30.5	20%-45%
Monocyte %	8.8	2%-10%
Eosinophil %	0.3	0%-6%
Basophil %	0.4	0%-2%
Immature granulocytes %	1.2	<2.0%
Nucleated red blood cells	0.0	0%

**Fig. 2:**
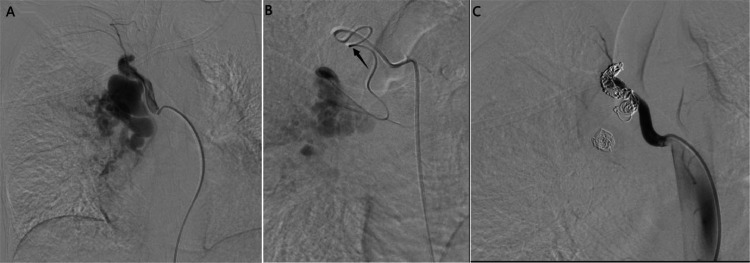
(A) Pre-embolization Fluoroscopic angiography (with contrast) confirming right bronchial artery aneurysm (2.3 × 3.6 cm). (B) Fluoroscopic angiography (with contrast) showing Successful distal catheterization using a Progreat microcatheter (2.7F) (black arrow) and embolization with Contour particles and coils. (C) postembolization Fluoroscopic angiography (with contrast) showing complete embolization with preserved flow in unaffected vessels.

The patient was then taken to the interventional radiology suite for angiographic evaluation. In a supine position, under an aseptic technique and local anesthesia, the right common femoral artery was punctured using an 18-gauge needle under ultrasound guidance. A 5 French (F) femoral introducer sheath was placed. Using a 0.035×150 cm angled Terumo wire and a C2 catheter, were used to successfully engage the right bronchial artery. Angiography showed a large, tortuous right bronchial artery with a segment of aneurysmal dilation. We advanced a Progreat microcatheter (2.7F) distal to the aneurysm and initiated embolization with Contour 710–1000 micron particles followed by multiple detachable embolization coil (Concerto™, Medtronic, USA) placements. A backdoor-frontdoor embolization approach was utilized to achieve complete occlusion. The final angiogram showed successful embolization with preserved flow in unaffected vessels (Fig. 2A-C).

After the BAE procedure, the patient remained stable and tolerated the procedure well. She resumed a regular diet and was discharged on the second-day postadmission. Close follow-up with imaging was recommended to monitor for any potential recurrence or complications.

## Discussion

Bronchial artery aneurysms (BAAs) are rare vascular anomalies representing abnormal dilatation of the bronchial artery [7]. The bronchial arteries, which are the main nutritional suppliers of lung tissue, most commonly arise from the descending thoracic aorta or a common intercostal artery trunk but can also originate aberrantly from the aortic arch, subclavian artery, internal mammary artery, intercostal arteries, or even coronary arteries [8]. BAAs exhibit significant variation based on their origin, which can influence clinical presentation and management [25,26]. For example, aneurysms originating from the subclavian or internal mammary artery may present with atypical chest pain, while those arising from the intercostal arteries can have a higher risk of spinal ischemia due to shared vascular supply [[27], [28], [29]]. In our case, the bronchial artery aneurysm originated directly from the thoracic aorta, which is among the most common sites and carries a risk of massive hemoptysis [30]. Despite their rarity (<1% of cases in selective bronchial arteriography), BAAs require prompt recognition and management due to their potential for life-threatening complications [7].

The reported cases of bronchial artery aneurysm were most commonly associated with definite etiologies that increase the shearing forces of the artery and weaken its wall, such as atherosclerosis or inflammatory lung disease, bronchiectasis, trauma, and sepsis (mycotic aneurysm) [[8], [9], [10], [11], [12]]. Other reports have described BAAs associated with systemic vascular abnormalities, such as Osler-Weber-Rendu and Behçet's disease [12]. However, there were some rarely reported cases without a clear etiology or associated morbidities [13], just like our case, a 31-year-old female who presented with a right bronchial artery aneurysm without known acute or chronic events or associated diseases.

Bronchial artery aneurysms manifest in various ways, most commonly presenting as hemoptysis. Other possible manifestations include chest pain, dyspnea, or symptoms related to mediastinal compression. They may also be incidentally discovered on imaging in asymptomatic patients [13]. A high suspicion is needed to make the diagnosis, especially in patients with idiopathic etiology, to avoid potential, life-threatening aneurysmal rupture and subsequent pulmonary hemorrhage. Various imaging modalities can aid in diagnosis, starting with a chest X-ray showing nonspecific abnormal shadows that prompt a confirmatory study for diagnosis [[14], [15]]. A CT scan of the chest with contrast is the commonly used modality for confirmation. Characteristic findings of the right bronchial artery may appear as dots or lines of increased density in the retro-tracheal, retro-bronchial, and retro-esophageal spaces [13,16], and in cases where a CT scan is nonuseful, magnetic resonance imaging and CT angiography of the chest can provide the needed diagnosis [16]. Our patient underwent a CT scan of the chest, which showed a dilated and tortuous right bronchial artery arising from the descending aorta with a few closely related dilated vascular structures. She then underwent a right bronchial artery angiogram for further evaluation.

Because rupture can be life-threatening, BAAs should be treated once the diagnosis is confirmed, whether there are symptoms or not [13]. Currently, transcatheter artery embolization is considered the first-line treatment for bronchial artery aneurysms due to its effectiveness, as reported in many previous cases. It is a safer approach with fewer complications and shorter recovery compared to traditional surgical intervention, which includes thoracotomy and surgical resection of the aneurysm; this approach is mainly used in ruptured aneurysms [13,17,18]. Different embolic materials have been used for BAE, with coils considered the first choice for bronchial artery embolization [8,18]. In our patient, A detachable embolization coil (Concerto™, Medtronic, USA) was chosen because this type of coils provide more precise, targeted occlusion, especially in aneurysmal or tortuous vessels. Contour particles, though effective, carry a higher risk of nontarget embolization, especially in cases with collateral circulation [8,18]. The combined use of coils and Contour particles ensured complete occlusion while minimizing complications [8,18].

Although rare, complications have been reported after bronchial artery embolization [2], ranging from mild, such as back pain, chest pain, and dysphagia, to more serious ones, such as vascular injuries (pseudo-aneurysm, dissection, or vessel perforation), stroke, nontarget embolization, and spinal cord ischemia [8,[19], [20], [21], [22], [23], [24]]. In our patient, the final angiogram showed a good result, her postembolization course was uncomplicated, and she was discharged the day after embolization.

In terms of outcomes, bronchial artery embolization had a success rate of 93.1% [18]. However, invasive follow-up by CT angiography is necessary to avoid any potential complications or unsuccessful embolization that mainly occurs due to aneurysm revascularization by collateral arteries connected with patent efferent bronchial arteries [7,8,12,18].

## Conclusion

This case highlights the importance of considering bronchial artery aneurysm in differential diagnoses. Despite its rarity, prompt diagnosis and treatment are crucial to preventing fatal complications. Early recognition and a multidisciplinary approach including interventional radiology for bronchial artery embolization were key to this patient's recovery and crucial in preventing mortality in these patients. Our successful embolization case highlights the critical role of coil-based bronchial artery embolization in managing BAAs. However, patients require follow-up with imaging after embolization to ensure there is no recurrence of bleeding or persistent perfusion of the aneurysm due to revascularization through collateral vessels.

## Ethics approval

Our institution does not require ethical approval for reporting individual cases or case series.

## Patient consent

I confirm that I have obtained written informed consent from the patient for the publication of this case report, including the use of her clinical details and radiological images. She has been informed that all efforts will be made to ensure anonymity, and no identifying information will be disclosed. She understands that the publication is for educational and scientific purposes and has agreed to its use.
